# Nationwide Prevalence of Groin Hernia Repair

**DOI:** 10.1371/journal.pone.0054367

**Published:** 2013-01-14

**Authors:** Jakob Burcharth, Michael Pedersen, Thue Bisgaard, Carsten Pedersen, Jacob Rosenberg

**Affiliations:** 1 Centre for Perioperative Optimization, Department of Surgery, Herlev Hospital, University of Copenhagen, Herlev, Denmark; 2 National Centre for Register-based Research, University of Aarhus, Aarhus, Denmark; 3 Department of Surgery, Køge Hospital, University of Copenhagen, Køge, Denmark; University of Michigan, United States of America

## Abstract

**Introduction:**

Groin hernia repair is a commonly performed surgical procedure in the western world but large-scaled epidemiologic data are sparse. Large-scale data on the occurrence of groin hernia repair may provide further understanding to the pathophysiology of groin hernia development. This study was undertaken to investigate the age and gender dependent prevalence of groin hernia repair.

**Methods:**

In a nationwide register-based study, using data from the Civil Registration System covering all Danish citizens, we established a population-based cohort of all people living in Denmark on December 31^st^, 2010. Within this population all groin hernia repairs during the past 5 years were identified using data from the ICD 10^th^ edition in the Danish National Hospital Register.

**Results:**

The study population covered n = 5,639,885 persons. During the five years study period 46,717 groin hernia repairs were performed (88.6% males, 11.4% females). Inguinal hernias comprised 97% of groin hernia repairs (90.2% males, 9.8% females) and femoral hernias 3% of groin hernia repairs (29.8% males, 70.2% females). Patients between 0–5 years and 75–80 years constituted the two dominant groups for inguinal hernia repair. In contrast, the age-specific prevalence of femoral hernia repair increased steadily throughout life peaking at age 80–90 years in both men and women.

**Conclusion:**

The age distribution of inguinal hernia repair is bimodal peaking at early childhood and old age, whereas the prevalence of femoral hernia repair increased steadily throughout life. This information can be used to formulate new hypotheses regarding disease etiology with regard to age and gender specifications.

## Introduction

Groin hernias can be classified according to several different classification systems [1] and is usually used to describe inguinal hernia (medial/direct and lateral/indirect) and femoral hernia in common. Groin hernia repair is a commonly performed general surgery procedure in both adults and children [1], [2] with inguinal hernias constituting more than 95% of all groin hernia repairs [3], [4]. Several hypotheses regarding the etiology of inguinal and femoral hernia have been proposed; however, very few studies address the epidemiologic aspects of inguinal and femoral hernia repair on a large scale level. Large scale prevalence data may facilitate new pathophysiological hypotheses, since a demographic overview of the disease is achieved.

This present study was undertaken with the primary objective to investigate the prevalence of inguinal and femoral hernia repair stratified into age and gender on a nationwide basis, which might form the basis for future more detailed studies.

## Methods

We established a register-based cohort consisting of all residents living in Denmark on December 31, 2010 using data from the Danish Civil Registration System [5]. The study period comprised a five year observation interval from January 1^st^, 2006 to December 31^st^, 2010. Within this time period all groin hernia repairs (inguinal and femoral hernia) were identified through the 10^th^ edition of the International Classification of Diseases retrieved from the Danish National Hospital Register [6]. We included all ICD-10 procedure codes for inguinal hernia repair: KJAB00, KJAB10, KJAB11, KJAB20, KJAB30, KJAB40, KJAB96, KJAB97 and femoral hernia repair: KJAC10, KJAC11, KJAC30, KJAC40, KJAC96, KJAC97, see Table 1.

**Table 1:** Surgical characteristics of the inguinal and femoral hernia procedures.

Inguinal hernia repair	Females	Males	Total
	4,455	41,109	45,564
KJAB00 (unspecified)	1,362	5,262	6,624
KJAB10 (plasty)	144	648	792
KJAB11 (laparoscopic)	921	6,375	7,296
KJAB20 (fascia transplant)	5	19	24
KJAB30 (mesh)	1,940	28,517	30,457
KJAB40 (plasty through laparotomy)	11	34	45
KJAB96 (other type)	53	121	174
KJAB97 (laparoscopic other type)	19	133	152
**Femoral hernia repair**	**992**	**421**	**1,413**
KJAC10 (unspecified)	316	124	440
KJAC11 (laparoscopic)	216	109	325
KJAC30 (mesh)	355	144	499
KJAC40 (plasty through laparotomy)	39	15	54
KJAC96 (other type)	60	24	84
KJAC97 (laparoscopic other type)	6	5	11

Both the Danish ICD-10 operative codes and a brief description of the procedures are mentioned.

The National Hospital Register contains information on all admissions to hospitals and surgical procedures performed since 1977. Since the National Hospital Register is the basis for economic reimbursement for medical services, there is a vested interest by providers to enter accurate information. The Civil Registration System was established in 1968 and contains information on all persons who were alive in Denmark in 1968 and forward. Among other variables, it contains information on the unique personal identifier number, gender, date and country of birth as well as current vital status. The unique personal identifier number is used as a personal identifier which enables accurate record linkage across all Danish national registers. This study included all acute, elective and ambulatory groin hernia repairs, however we were not able to discriminate between them due to register limitations.

For each person in the study population every inguinal and femoral hernia repairs were identified trough the National Hospital Register during the five year study period. Each person could appear in both the inguinal and femoral hernia repair groups, however it was only possible to appear with one repair (the first) in each of the hernia groups, no matter the amount of repairs performed. The inguinal hernia repairs were subdivided into two year intervals 0–2, 2–4, 4–6 years etc. and femoral hernia repairs into ten year intervals 0–10, 10–20, 20–30 years etc. The reason for this specific subdivision was the substantially larger inguinal hernia patient material.

## Statistics and Permissions

The prevalence estimates were calculated as the number of people operated for either inguinal or femoral hernia during the five year study divided by the number of citizens living in Denmark on December 31^st^, 2010. The prevalence estimates indicated the percentage of the corresponding age group of the population that at a given time during the five year period had a groin hernia repair performed. These analyses were stratified by gender and age. The 95 percent likelihood ratio based confidence limits for the five year prevalences were estimated by binomial regression using the logarithm as offset [7].

The study was approved by The Danish Data Protection Agency no. 2011-41-6149 and the Danish National Board of Health no. 7-505-29-1765/1. The study was not evaluated by a local ethics committee, since only biomedical studies require ethic committee approval according to Danish law.

## Results

**Figure 1:**
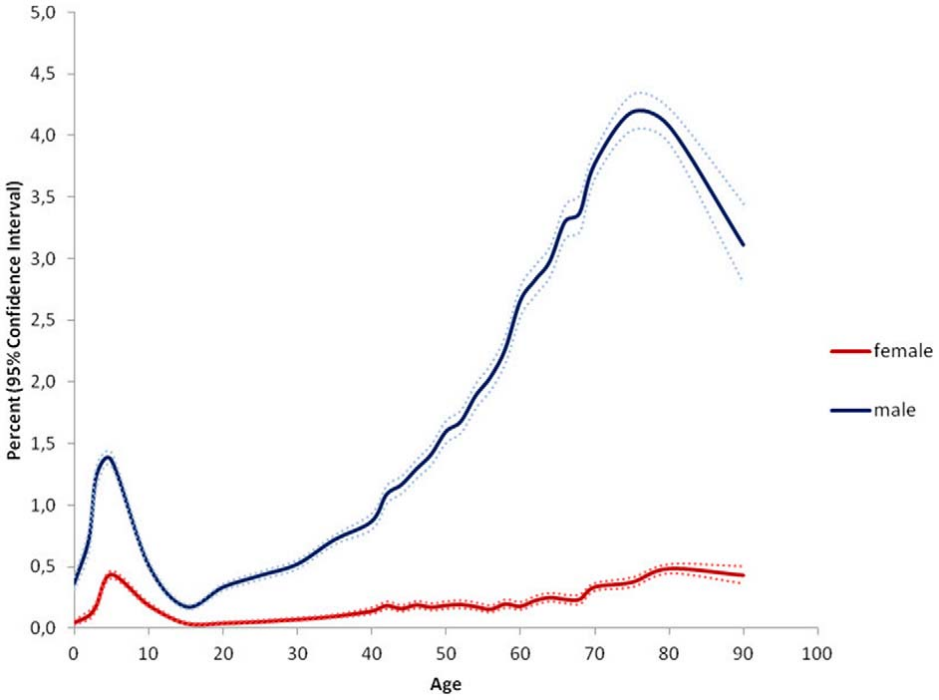
Prevalence of groin hernia repair, inguinal hernia and femoral hernia combined, stratified by age and gender. The results indicate the percentage of persons at a given age in the population who were operated for a groin hernia during the study period. Example: 4.19% CI 4.04–4.34% of all males aged 75–80 years in Denmark were operated for a groin hernia at least once during the study period.

**Figure 2:**
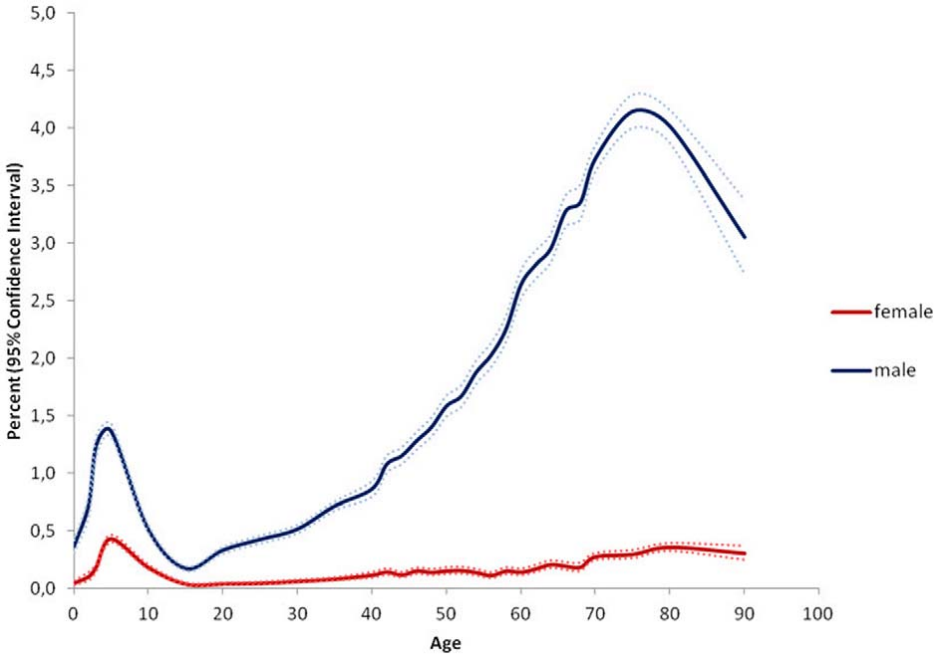
Prevalence of inguinal hernia repair stratified by age and gender. The results indicate the percentage of persons at a given age in the population who were operated for an inguinal hernia during the study period. Example: 4.14% CI 4.0–4.29% of all males aged 75–80 years in Denmark were operated for an inguinal hernia at least once during the study period.

**Figure 3:**
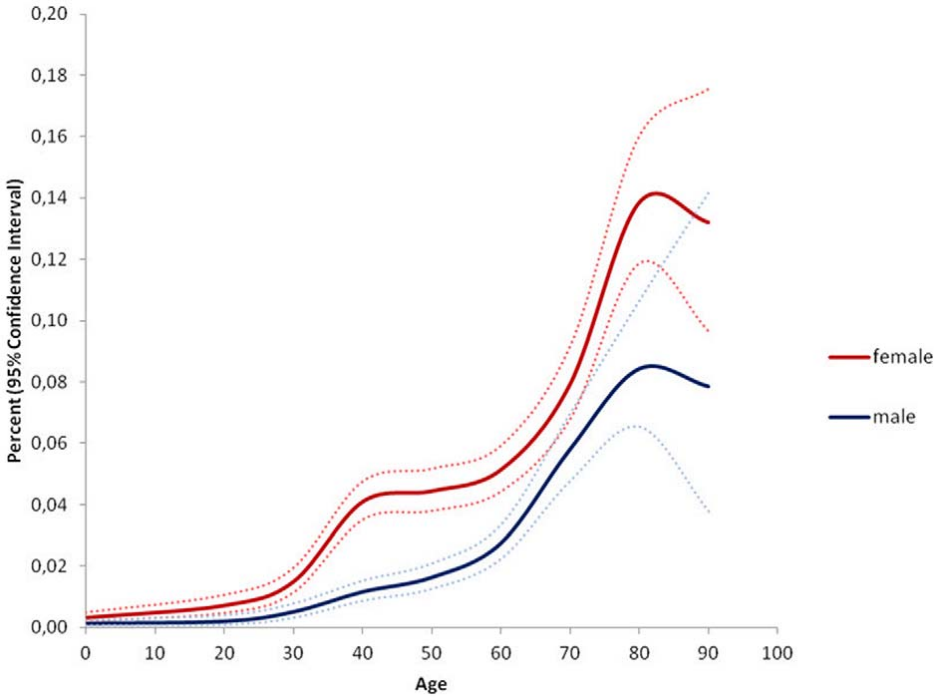
Prevalence of femoral hernia repair stratified by age and gender. The results indicate the percentage of persons at a given age in the population who were operated for a femoral hernia during the study period. Example: 0.14% CI 0.12–0.16% of all females aged 80–90 years in Denmark were operated for a femoral hernia at least once during the study period.

**Figure 4:**
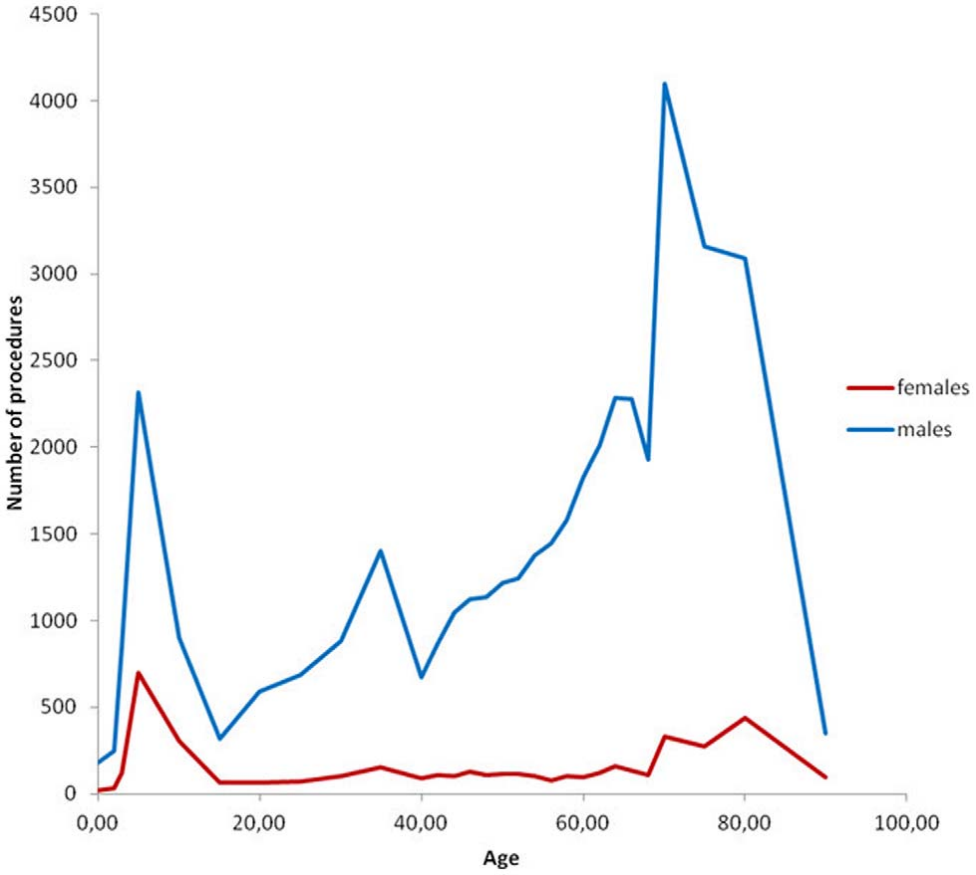
Numerical number of inguinal hernia repairs performed stratified by age and gender.

**Figure 5:**
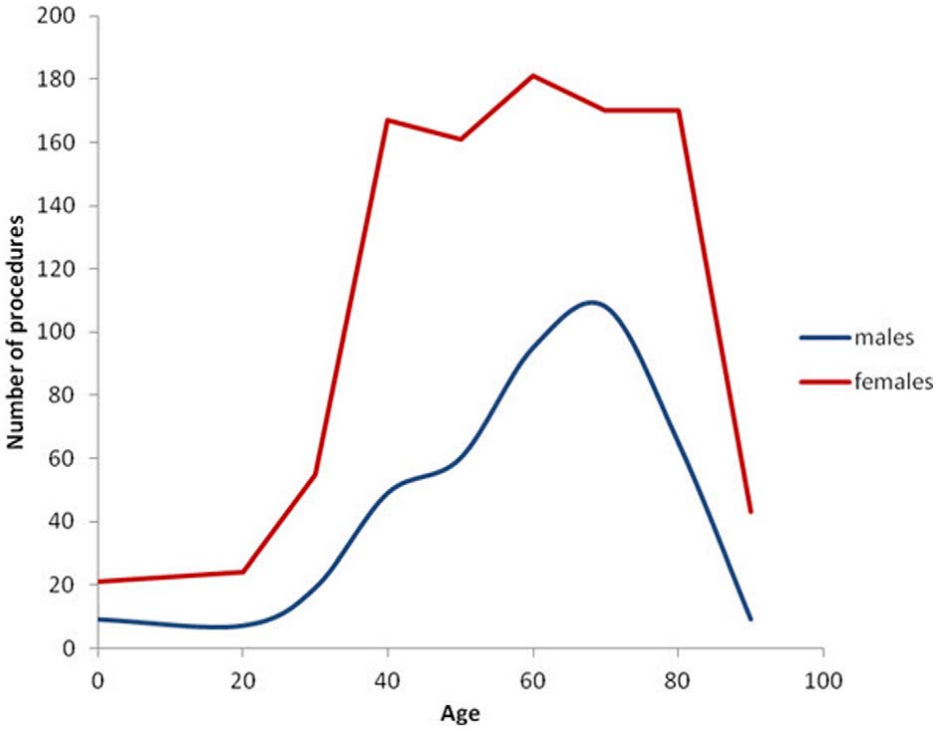
Numerical number of femoral hernia repairs performed stratified by age and gender.

The study population covered n = 5,639,885 persons including 2,799,105 males and 2,840,780 females. Within this population 46,717 persons, 88.6% males and 11.4% females, had at least one groin hernia repair during the five year study period, see Figure 1. Surgical characteristics of the performed procedures are mentioned in Table 1. Inguinal hernias comprised 97% of groin hernia repairs, 90.2% males and 9.8% females, and femoral hernias 3% of groin hernia repairs with 29.8% males and 70.2% females, see Figure 2 and Figure 3. Measured in the total amount of procedures, the overall inguinal to femoral hernia repair ratio was 32∶1, i.e. 32 times more inguinal hernia repairs were performed compared with femoral hernia repairs. The actual numbers of inguinal and femoral hernia repairs performed are shown in Figure 4 and Figure 5, respectively.

Especially in men, however also in women the age-specific prevalence of inguinal hernia repair showed a bimodal distribution. The peak-prevalence in childhood in the age group 0–5 years was 0.43% CI 0.40–0.46% in females and 1.36% CI 1.31–1.42% in males, whereas the peak-prevalence in old age in the age group 75–80 years was 0.36% CI 0.32–0.39% in females and 4.14% CI 4.0–4.29% in males, see Figure 2. Inguinal hernia repair prevalence was higher for males than females in all age groups.

The age-specific prevalence of femoral hernia repair increased steadily throughout life with a peak-prevalence at the age of 80–90 years in 0.14% CI 0.12–0.16% of females and 0.08% CI 0.07–0.11% of males, see Figure 3. Inguinal hernia repair was more commonly performed in childhood in comparison with femoral hernia repair in both genders. Only 2% of the total number of females and males with a femoral hernia repair were performed before the age of 20 years, whereas 30% of all females with an inguinal hernia repair and 12% of all males with an inguinal hernia repair were performed before the age of 20 years.

## Discussion

This nationwide register-based study provided large scale evidence for age- and gender distribution of groin hernia repairs. Inguinal hernia repair showed a bimodal peaking prevalence at early childhood and old age for both genders, however more pronounced in males than females. Males had a higher inguinal hernia repair prevalence compared with females at all ages. Femoral hernia repair prevalence increased steadily with age throughout life in both genders, and in all ages females had higher femoral hernia repair prevalence compared with males.

Our study confirmed the existing literature in the fact that inguinal hernia repair constituted the vast majority of groin hernia repairs in both males and females compared with femoral hernia repair [8]–[11]. Furthermore, these results underline that inguinal hernias primarily affect males and femoral hernias primarily affect females. The results question the normal understanding that primarily elderly women are operated for a femoral hernia, see Figure 3. Even though femoral hernia repair occurred more commonly among females than males, the most common female groin hernia was the inguinal hernia, which outnumbered the female femoral hernia repair frequency five times. Thus, a lump in the groin in a female most likely is an inguinal hernia until proved otherwise. Regarding children's groin hernia, our data showed that femoral hernias were almost absent in childhood, see Figure 3. Thus, our findings support that a groin hernia in a child most likely is an inguinal hernia, corresponding with the literature [12].

Earlier studies investigating epidemiologic aspects of groin hernia repair in the western world are few and heterogeneous [13]–[16]. A series of clinical studies examining industrial workers and males registered for military service found groin hernia prevalence ranging from 2–9% [13], [14], [16]. All of these studies relied on clinically groin hernia diagnosis performed by several examiners and included demographic restrictions of age and gender, which could reduce the possibility of transferring the results to a general population. A clinical study examining inguinal hernias in males aged 25–75 years found prevalence estimates ranging from 1–30% [15]. These prevalence estimates seem high compared with our results, but could be due to design circumstances such as population size, method of clinical examination and diagnostic criteria. The only earlier population-based inguinal hernia study generally reported lower prevalence estimates compared with our data [17]. They found an inguinal to femoral hernia ratio of 16∶1 which is notably lower than our results, and furthermore a male to female ratio of 8∶1 was reported [17]. A possible explanation of the low inguinal to femoral hernia ratio could be the use of many different non-surgical examiners rather than basing hernia data on surgery information, which may have increased the amount of falsely diagnosed groin hernias. However, it should be observed that overall comparison of gender-ratios between different studies should be reserved to situations where the gender ratio is identical for all age-groups, which is not the case for neither inguinal hernias nor femoral hernias. Therefore, we have chosen not to mention comparative gender-ratios and the reader should consider only the full shape of the five year prevalences shown in the Figures 1, 2, 3.

Some weaknesses and limitations exist in this study. The prevalence estimates given in this study are an approximation of the true groin hernia prevalence in the population, since data exclusively were based on groin hernia repairs and did not include groin hernias diagnosed by clinical examination or groin hernias evaluated not eligible for operation. Obviously, some patients are not eligible for hernia repair or do not seek medical help for their hernia, and it has earlier been shown that a watchful waiting strategy among males with inguinal hernias and minimal symptoms can be recommended [18]. By basing results on number of hernia operations rather than number of clinical diagnosed hernias, we report data on patients with hernias who were expected to benefit from treatment. We could not discriminate between primary or recurrent procedures since the ICD-10 classification does not allow separation between these. This will to a lesser extent have influenced the childhood prevalence estimates, since most procedures performed in children assumedly were primary procedures. However, the prevalence estimates rates in the elderly part of the study population are most likely higher than if we had had the possibility to focus solely on primary procedures. This was attempted reduced by restricting the same person from appearing more than once in each of the hernia categories inguinal and femoral hernia, regardless the number of procedures the person had undergone in the study period. The ICD-10 classification does not allow discrimination between repairs for medial/direct or lateral/indirect inguinal hernias, which otherwise would have been interesting since it has been shown that the majority of inguinal hernias in childhood are lateral, whereas adult inguinal hernias are a mixture of lateral and medial [2], [19]. It is not possible to estimate the life-time risk of groin hernia repair based upon prevalence data. Only by using incidence data it is possible to comment on when persons are operated for their first groin hernia, which is necessary in order to approximate life time risk.

## Conclusion

This nationwide prevalence study showed that the age distribution of inguinal hernia repair is bimodal peaking at early childhood and old age, whereas the prevalence of femoral hernia repair increased steadily throughout life. This information can potentially create a basis for formulating new hypotheses regarding disease etiology.
